# Ethanol induces cell-cycle activity and reduces stem cell diversity to alter both regenerative capacity and differentiation potential of cerebral cortical neuroepithelial precursors

**DOI:** 10.1186/1471-2202-6-59

**Published:** 2005-09-13

**Authors:** Daniel R Santillano, Leena S Kumar, Terasa L Prock, Cynthia Camarillo, Joseph D Tingling, Rajesh C Miranda

**Affiliations:** 1 Department of Human Anatomy & Medical Neurobiology, Texas A&M University System Health Science Center, College of Medicine, College Station, TX, USA; 2 Centre for Environmental and Rural Health, Texas A&M University, College Station, TX, USA

## Abstract

**Background:**

The fetal cortical neuroepithelium is a mosaic of distinct progenitor populations that elaborate diverse cellular fates. Ethanol induces apoptosis and interferes with the survival of differentiating neurons. However, we know little about ethanol's effects on neuronal progenitors. We therefore exposed neurosphere cultures from fetal rat cerebral cortex, to varying ethanol concentrations, to examine the impact of ethanol on stem cell fate.

**Results:**

Ethanol promoted cell cycle progression, increased neurosphere number and increased diversity in neurosphere size, without inducing apoptosis. Unlike controls, dissociated cortical progenitors exposed to ethanol exhibited morphological evidence for asymmetric cell division, and cells derived from ethanol pre-treated neurospheres exhibited decreased proliferation capacity. Ethanol significantly reduced the numbers of cells expressing the stem cell markers CD117, CD133, Sca-1 and ABCG2, without decreasing nestin expression. Furthermore, ethanol-induced neurosphere proliferation was not accompanied by a commensurate increase in telomerase activity. Finally, cells derived from ethanol-pretreated neurospheres exhibited decreased differentiation in response to retinoic acid.

**Conclusion:**

The reduction in stem cell number along with a transient ethanol-driven increase in cell proliferation, suggests that ethanol promotes stem to blast cell maturation, ultimately depleting the reserve proliferation capacity of neuroepithelial cells. However, the lack of a concomitant change in telomerase activity suggests that neuroepithelial maturation is accompanied by an increased potential for genomic instability. Finally, the cellular phenotype that emerges from ethanol pre-treated, stem cell depleted neurospheres is refractory to additional differentiation stimuli, suggesting that ethanol exposure ablates or delays subsequent neuronal differentiation.

## Background

Children exposed to alcohol during gestation can exhibit a spectrum of abnormalities that range from Alcohol Related Neurodevelopmental Disorders (ARND) to Fetal Alcohol Syndrome (FAS), based upon the severity of symptoms. These abnormalities can include facial anomalies, growth deficits, mental retardation, attention deficit/hyperactivity disorders, motor difficulties, learning and memory impairment and psychological disorders such as depression [1-9]. Ethanol is teratogenic and exerts pleiotrophic effects in the differentiating nervous system, including induction of cell-death mechanisms [10-13], disruption of trophic support [14-17] and deregulation of neurotransmitter networks like the GABA, glutamate and serotonergic systems [18-24].

During prenatal period of neurogenesis, the number of neuroepithelial cells expands rapidly to generate most of the neurons of the adult brain [25] requiring, as with other tissues [26], the conversion of un-committed stem cells to more fate-restricted neuroblasts, and ultimately neurons. Ethanol exposure during gestation may alter the number and types of neuronal stem and blast precursors available for normal development, potentially producing irreversible damage to the developing brain. For example, previous research using BrdU incorporation analyses has shown that ethanol suppresses cell-cycle in the ventricular zone while promoting proliferation in the more mature subventricular zone [27]. The opposing effect of ethanol on these two cell populations indicates that ethanol disrupts the normal balance of precursor populations and suggests that ethanol may not affect all immature precursors similarly. We know little about the molecular heterogeneity of cortical neuroepithelial cells, though emerging evidence suggests that the neuroepithelium is quite heterogeneous with respect to differentiation and gene expression states of its constituent cells [28-30]. Cell surface markers like CD133/prominin-1, Sca-1 (Ly6A/E), CD117/c-kit and ABCG2 (ATP-binding cassette, sub-family G (WHITE), member 2) have been used successfully to monitor stem cell heterogeneity in a variety of tissues [31-34], and we therefore used these markers to monitor neuronal stem cell heterogeneity following ethanol exposure. We hypothesized that if ethanol influenced the proliferation of neuroepithelial cells, it would also alter the numbers of stem cells within the cortical neuroepithelium.

Finally, mechanisms that maintain genomic stability are important during neurogenesis because the frequency of DNA synthesis errors and aberrant chromatin assembly are increased during periods of robust proliferation. These errors must be limited, so that neural stem cells do not accumulate and transmit genetic damage to daughter cells. The telomerase complex, a reverse transcriptase enzyme complex, maintains telomeres during DNA replication [35-37], thereby preventing genomic instability and cellular senescence. Telomerase is active in all germline tissues, transformed cells and most human cancers [38,39], but is particularly robust during neurogenesis where it has been shown to function as an anti-apoptotic factor in developing neurons [40,41]. The activation of telomerase during neurogenesis, and the anti-apoptotic role of telomerase in neurons, led us to hypothesize that telomerase is a molecular target for ethanol during neuroblast expansion.

To examine the effects of ethanol on cortical precursor growth and survival, neurosphere cultures were generated from gestational day 15 rat fetuses. Contrary to our initial hypothesis, prolonged ethanol exposure promoted cell-cycle activity without concomitant increases in telomerase activity, or significant apoptosis. However, despite directly increasing cell cycle activity, ethanol suppressed expression of several stem cell markers, and decreased the future proliferation and differentiation potential of exposed cortical-derived neurospheres. Collectively, these data indicate that stem cell diversity, stability, and maturation are likely to be important components of the prenatal brain damage caused by maternal ethanol consumption.

## Results

**Figure 1:**
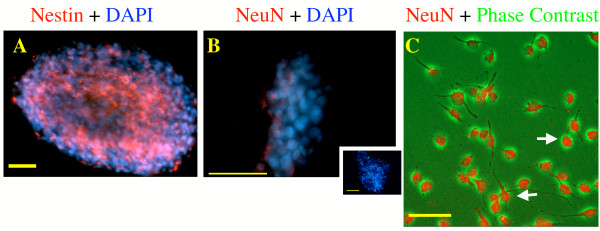
Photomicrographs depicting immunofluorescence analysis of neurospheres labeled for intermediate filament proteins nestin (**A**), and the neuronal lineage marker, the neuronal-specific nuclear antigen, NeuN (**B **and lower magnification inset). Cell nuclei were counter-stained with DAPI (blue). Control neurosphere cultures are immuno-positive for nestin. However, cultures do not exhibit nuclear localization of NeuN, showing that neurosphere cultures were comprised of immature cells. (**C**) Differentiation following removal of the mitogen EGF, and dispersion of cells onto a laminin substrate, results in the upregulation and nuclear localization of NeuN. Photomicrograph represents a digitally merged immunofluorescence and phase contrast microscopic image showing NeuN immunofluorescence overlying nuclei (white arrows) of early differentiating neurons. Scale bars: 50 uM.

**Figure 2:**
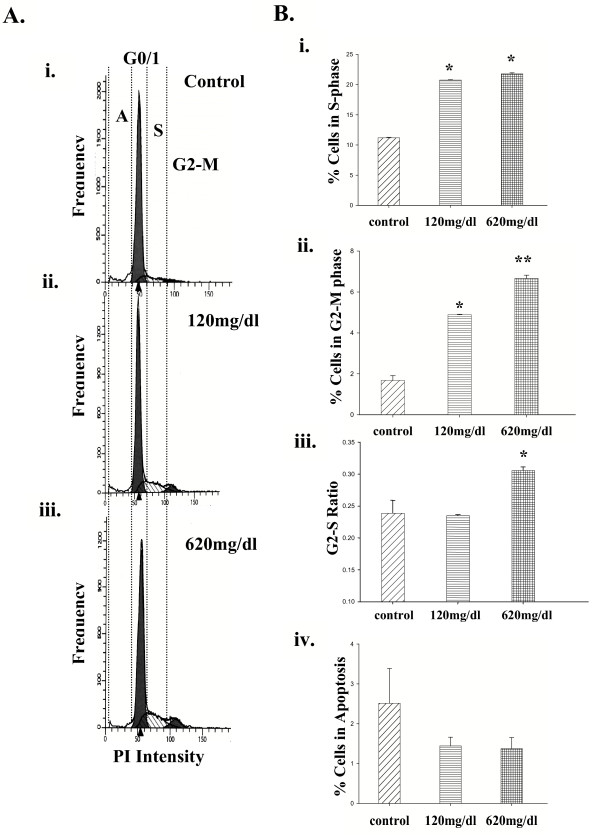
(**A**) Cell-cycle analysis of cortical progenitors treated with ethanol for 4 days. (**A*i-iii***) Flow-cytometric frequency histograms of progenitors stained with propidium iodide (PI) for DNA content. (**A*ii,iii***) Ethanol stimulates DNA synthesis and cell-cycle progression, as indicated by the increase in area under the S-phase and G2/M peaks, relative to controls. **B*i-iii***, Quantitative analyses of cell-cycle. (**B*i,ii***) Ethanol significantly increased the number of cells entering S and G2/M-phases of the cell-cycle. (**B*iii***) The G2/S ratio was unchanged at low ethanol doses, but significantly increased with the high dose. (**B*iv***) Ethanol did not induce apoptosis at either dose used, and very little DNA fragmentation was observed in the sub-G0/1 range in (***Ai-iii***). Asterisks indicate statistical-significance, p < 0.05.

Neuronal progenitor cells, including multi-potent stem, and more committed blast cells, can be isolated from the developing cortex, expanded *in-vitro *as neurosphere aggregates, and subsequently used to generate neurons and glia [42-44]. Acutely dissociated cortical progenitors were cultured as neurosphere aggregates in serum-free mitogenic media to model the period of neuroblast precursor expansion during neurogenesis in the rodent. Cultured neurospheres were immuno-positive for nestin (Figure 1A), consistent with the hypothesis that neurospheres were comprised of immature cells. In contrast, immunofluorescence for the neuronal nuclear antigen (NeuN) was expressed within the soma of a few progenitor cells at the periphery of the neurosphere, but did not co-localize to any nucleus (Figure 1B). The presence of nestin [45] indicates that neurospheres are immature and multi-potent, and the lack of nuclear labeling for NeuN (Figure 1B) indicates that these cells are not yet committed to neuronal differentiation. In contrast, differentiation, induced by removal of Epidermal Growth Factor and culturing neurosphere-derived cells on a laminin substrate, leads to clear nuclear expression of NeuN (Figure 1C) and suppression of nestin immunoreactivity (data not shown), suggesting that neurosphere-derived cells can be committed to a neuronal lineage. Additionally, our flow cytometric data (Figure 2) indicate that, on average, less than 4% of cells in neurosphere cultures are apoptotic, suggesting that a majority of cells are viable in this culture model.

### Ethanol exposure activates cell cycle activity in primary cortical neurosphere cultures and does not lead to apoptosis

Figure 2A (i-iii) illustrates the proportion of cells in cell cycle in response to ethanol treatment measured by flow cytometry. Exposure to ethanol for 4 days induced cell cycle activity at 120 mg/dl and 620 mg/dl, respectively. The proportion of cells in S-phase increased significantly (by 1.8–1.9 fold) after exposure to both doses of ethanol, relative to controls (Figure 2Bi). Furthermore, the ethanol-stimulated increase in the S-phase fraction was mirrored by a significant increase (1.8 – 2.5 fold) in the percentage of cells progressing to the G2 phase of the cell cycle (Figure 2Bii). The ratio of G2/S reflects the proportion of cells completing DNA synthesis and cellular division. At low ethanol concentrations (120 mg/dl), the G2/S ratio was similar to controls, but the ratio increased with higher ethanol concentrations (620 mg/dl) suggesting that the increase in DNA synthesis did result in progression to the G2 phase of the cell cycle (Figure 2Biii). Ethanol did not alter the number of cells with less than G0 DNA content (a marker of apoptosis, [46]), indicating that ethanol did not induce significant apoptosis in cortical-derived neurosphere cultures (Figure 2Biv).

**Figure 3:**
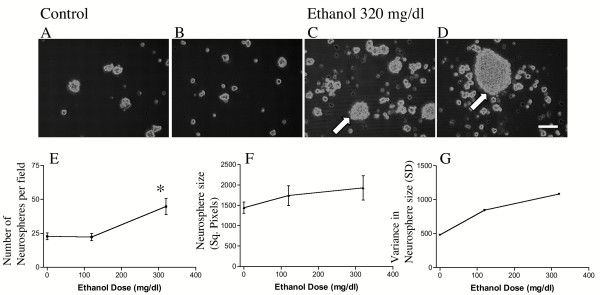
Ethanol increases neurosphere number and increases variation in the size of neurospheres. (**A-D**) Representative photomicrographs of control (**A,B**) and ethanol-treated (**C,D**) neurospheres showing that ethanol (at 5 days exposure) increases both the density and size variation of neurospheres. Arrows mark the appearance of large neurospheres in ethanol-treated cultures. (**E-G**) Morphometric analyses show that ethanol induces a significant increase in the density of neurospheres (**E**), without altering the mean area per neurosphere (in square pixels, **F**). However, ethanol induced a dose-related increase in variation (Standard Deviation) in neurosphere size (**G**). Asterisks indicate statistical-significance, p < 0.05. Scale bar: **A-D**, 100 uM.

Consistent with the above data, morphometric analyses indicate that ethanol significantly increased the density of neurospheres per field (Figure 3A–D). Analysis of variance followed by post-hoc statistical analyses indicated that ethanol induced a dose-related increase in the density of neurospheres (p < 0.001, Figure 3E). We observed a moderate but statistically significant correlation (Pearson product moment correlation (r) of 0.52 (p < 0.001)) between ethanol dose and density of neurospheres in the culture dish. When we controlled for the effect of neurosphere size on the density of neurospheres (i.e., the fact that an increase in the number of large neurospheres would effectively decrease the density of neurospheres within a field), the partial correlation coefficient between ethanol dose and neurosphere number increased (r = 0.71, p < 0.0001). While an overall analysis of variance indicated that ethanol did not statistically increase neurosphere size (Figure 3F), ethanol did lead to a ~2-fold increase in variation in neurosphere size at 120 mg/dl and a ~3-fold increase at 320 mg/dl within the culture dish, as indicated by an increase in the variance measure (i.e., Standard Deviation, Figure 3G). Therefore, ethanol-treated cultures exhibited a high degree of variability in neurosphere size compared to untreated cultures, and some of the neurospheres in ethanol treated cultures were extremely large (Figure 3C, D compared to 3A&B). It is possible that the ethanol-associated increased size and growth rate of neurospheres may translate into decreased viability of stem and progenitor pools within the neurosphere. However, this scenario is unlikely to be true since the overall rate of cell death was unchanged by treatment condition (Figure 2). Hence, these data collectively support the hypothesis that ethanol induced cell cycle in neurosphere cultures. One interesting observation was that ethanol-exposed neurospheres loose their spherical shape and exhibit irregular edges, suggesting either a structural disorganization of the neurosphere, perhaps due to variable growth rates within different parts of an individual neurosphere, or alternatively, the merging together of smaller neurospheres.

**Figure 4:**
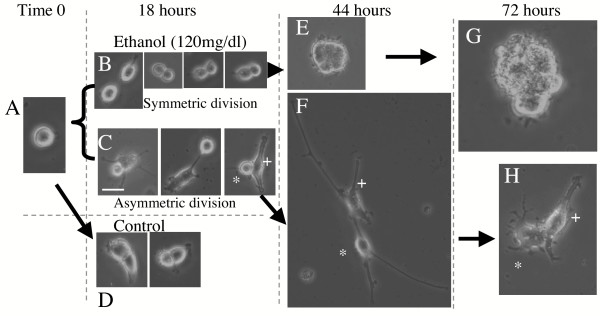
Ethanol induces asymmetric division of neural progenitor cells. Dissociated neural progenitor cells (**A**) cultured under mitogenic conditions divide symmetrically to generate two similar daughter cells (**D**, representative examples of cells undergoing cytokinesis), and regenerate new neurospheres (data not shown). Ethanol (120 mg/dl)-treated neural progenitors also generate daughter cells by symmetrical division (**B**, examples of cells in different stages of cytokinesis), to regenerate neurospheres over a period of 44 (**E**) and 72 (**G**) hours, under mitogenic conditions. However, despite being cultured under mitogenic conditions, ethanol-treated progenitors also exhibit asymmetric division to generate morphologically dissimilar daughter cells (**C**, examples of asymmetrical division, showing daughter cells in different stages of cytokinesis). Over the period of 44 to 72 hours, one daughter cell exhibits somatic mobility (e.g., **F,H**, cell marked by a *), while the second daughter cells remains stationary (e.g., **F,H**, cell marked by +). The motile daughter cell transiently exhibits long filamentous processes (**F**,*), though these processes retract over a period of 72 hours (**H**,*), in mitogenic medium.

Our observation that ethanol-treated neurospheres exhibited increased heterogeneity in size and assumed a disorganized shape over time suggested that ethanol may promote differential responses among cells within an individual neurosphere. We therefore dissociated neurospheres (Figure 4A), and cultured individual cells in mitogenic medium with or without ethanol (120 mg/dl) to determine if individual cortical precursors could regenerate neurospheres. Over a four-day treatment period, control (Figure 4D) and ethanol-treated neural progenitors (Figure 4B) proliferated symmetrically, to generate morphologically similar daughter cells (symmetrical division, Figure 4E), and ultimately to generate new neurospheres over a 72-hour period (Figure 4G). Under the mitogenic conditions used (see cell culture methods), we only observed symmetrical cell division in control cultures. However, in contrast to control cultures, ethanol-treated neurospheres also exhibited an asymmetric mode of division, where one daughter cell assumed a more differentiated morphology compared to its mitotic partner (Figure 4C). One of the asymmetrically dividing pair of cells tended to be non-motile, while the second daughter cell exhibited extensive somatic motility (Figure 4F, H) over a period of 72 hours. In a majority of cases, the more morphologically differentiated member of the asymmetrically dividing pair assumed a stellate morphology. Occasionally, one of the pair of asymmetrically dividing daughter cells transiently expressed elongated, radial-glia-like processes (e.g., Figure 4F), before assuming a stellate appearance, despite the continued presence of mitogenic medium.

**Figure 5:**
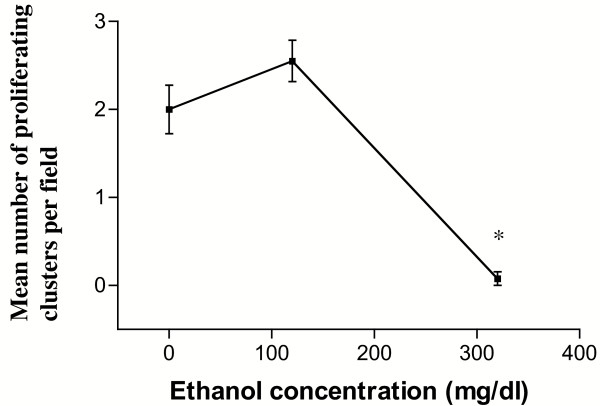
Quantitative analysis of the effect of ethanol-pretreatment on the regenerative capacity of neural progenitor cells. Neurosphere cultures were treated with varying doses of ethanol for five days, then dissociated and cultured as individual progenitor cells, in ethanol-free mitogenic medium. Ethanol pre-treatment leads to a dose-related decline in the subsequent ability of neural progenitors to undergo cell division (mean number of dividing cells per well ± SEM) and generate clonal colonies of cells. Asterisks indicate statistical-significance, p < 0.05.

The presence of asymmetric cell division events in ethanol-treated cultures suggested that ethanol forces progenitor cell maturation, potentially depleting the numbers of self-replicating progenitors in neurosphere cultures. Therefore, we next determined the extent to which ethanol altered the future proliferation capacity of neural progenitor cells. We treated neurospheres with ethanol for four days; then dissociated the neurospheres into single cells that were cultured in mitogenic medium for two days. Prior exposure to ethanol led to a statistically significant (p < 0.0001, Figure 5) dose-related decline in the numbers of clonal colonies that were formed from dissociated cells, suggesting that a prior episode of ethanol ultimately depletes the proliferative capacity of cortical progenitors.

### Ethanol suppresses cell-surface stem cell marker expression but not nestin mRNA expression in neurospheres

**Figure 6:**
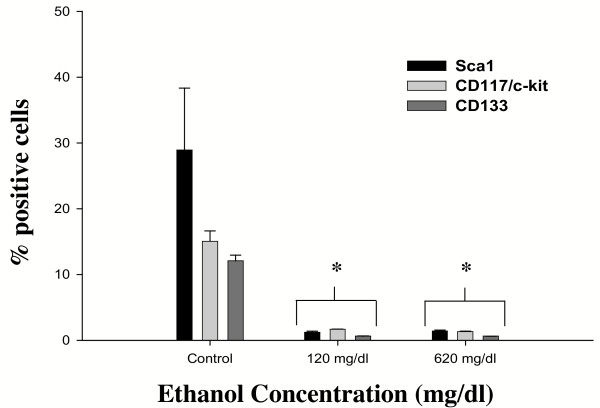
Quantitative analysis of flow-cytometric data for stem-cell antigen expression in cortical progenitors. Progenitors express stem-cell antigens *(control)*. After 4 days of treatment, low doses of ethanol significantly reduced the proportion of cells expressing Sca-1, CD117/C-kit and CD133. The high dose of ethanol did not further reduce stem-cell antigen expression. Asterisks indicate statistical-significance, p < 0.05. Data were collected from at least 10,000 events/sample.

Neurosphere cultures consist of a heterogeneous mixture of immature neuronal stem cells and more differentiated daughter neuroblasts [47]. Based on our observations that ethanol promoted cell cycle activity, we hypothesized that ethanol would also lead to an aberrant expansion of the stem cell pool in neurosphere cultures. In the initial set of experiments, we utilized flow-cytometry to examine the expression of three stem cell markers, Sca-1 (Ly6A/E), CD117/c-kit and CD133/prominin-1. Figure 6 shows that these stem cell antigens are expressed *in vitro *in control proliferating neurosphere cultures. However, contrary to our hypothesis, we observed a large and statistically significant decrease in the numbers of cells expressing Sca-1 (~23-fold), CD117/C-kit (~9-fold) and CD133/prominin-1 (~19-fold) on their cell surface, after treatment with ethanol at 120 mg/dl (p < 0.05, N = 9 samples per stem cell antigen group). Increasing the dose of ethanol did not lead to a further reduction in stem cell antigen expression.

**Figure 7:**
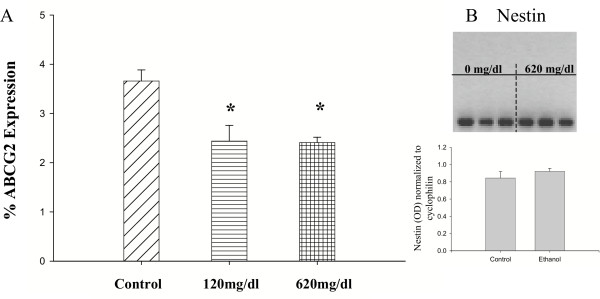
(**A**) ABCG2 Transporter expression in cortical progenitors after ethanol treatment. Cultures w ere exposed to ethanol and live, immuno-labeled cells were processed for FACS analysis (see methods) with antibodies to surface ABCG2. Ethanol significantly reduced the expression of ABCG2 (p < 0.05). Data were collected from 100,000 events/sample. (**B**) RT-PCR analysis for nestin in cortical progenitors treated with ethanol for 4 days. The levels of each transcript were unchanged by ethanol. Transcript levels were normalized to cyclophilin (data not shown).

We wanted to examine the extent to which ethanol suppression of cell-surface stem cell marker expression was a generalized phenomenon that could be observed with other, more selective, stem-cell markers. Therefore, in our next experiment we examined the expression of the ATP-binding multidrug transporter ABCG2, an integral membrane protein that identifies stem cells in many tissues including the nervous system [48], and is considered a reliable marker for stem cells [49-52]. We examined the cellular expression of ABCG2 by flow cytometry in neurosphere cultures exposed to ethanol at the same dosages and time period. Figure 7A shows that ethanol significantly reduced the number of live cells that expressed ABCG2 immunofluorescence after 4 days (p < 0.05). These data suggest that ethanol does indeed suppress the expression of a specific cell-surface stem cell marker in neurosphere cultures.

In contrast to the above cell-surface markers, nestin is an intermediate filament protein that is expressed by both stem and progenitor cells within neurospheres ([45] and Figure 1A), and levels decline in differentiated post-mitotic neurons [53]. We hypothesized that if ethanol suppressed the expression of cell-surface stem cell markers, it would similarly suppress the expression of mRNA for nestin. However, semi-quantitative RT-PCR analysis (Figure 7B) showed that there was no change in nestin mRNA expression (normalized to cyclophilin-A) following ethanol exposure. These data suggest that while ethanol may not decrease the overall size of the precursor pool (the combined stem and blast pool, indicated by constant nestin mRNA expression), it decreases the diversity of stem cells within this pool (indicated by decreased numbers of cells expressing Sca-1, c-kit, CD133 and ABCG2).

### Ethanol reduces telomerase reverse transcriptase (TERT) mRNA levels in neurosphere cultures

**Figure 8:**
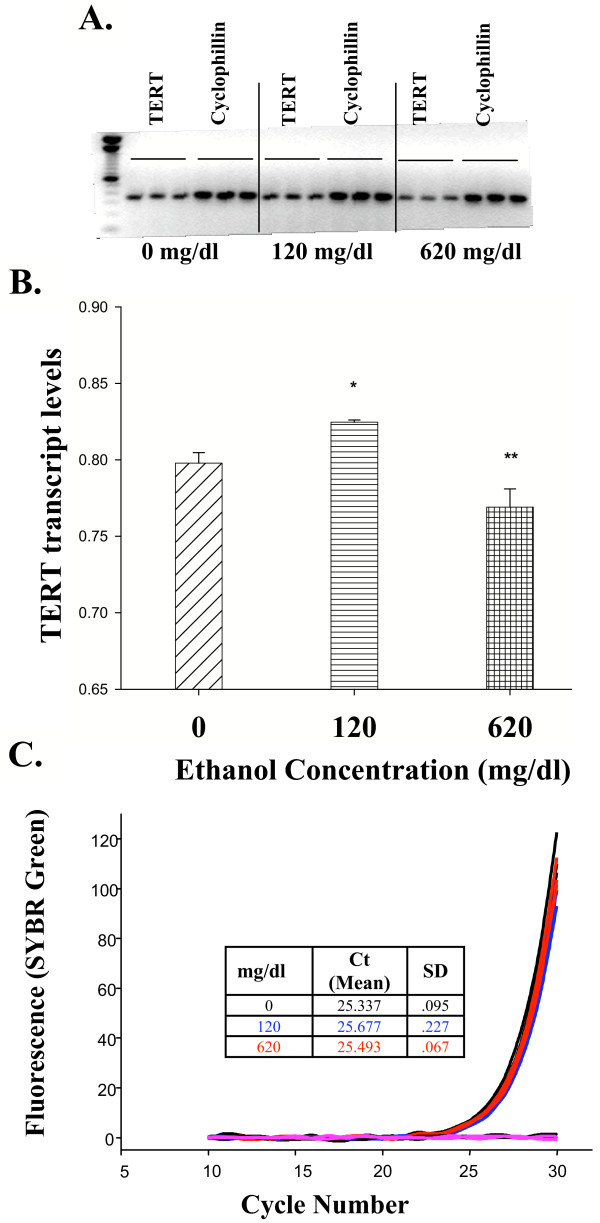
(**A,B**) TERT mRNA expression and telomerase activity in cortical-derived neurospheres treated with ethanol for 4 days. (**A**), RT-PCR for the TERT transcript. (**B**), Quantification of TERT levels in *A*, expressed as a ratio to cyclophilin. Low doses of ethanol modestly, but significantly, increased TERT transcript levels. However, high doses of ethanol decreased TERT mRNA expression. (**C**), TRAP assays revealed no differences in telomerase activity between treated and control groups. The cycle threshold value (Ct) indirectly measures activity; a lower Ct corresponds to higher telomerase activity. Inset table indicates Ct ± Standard Deviation (SD). Color code: Black, control; blue, 120 mg/dl; red, 620 mg/dl; pink, no-telomerase control. Asterisks indicate statistical-significance, p < 0.05.

Telomerase reverse transcriptase (TERT) has been shown by others to function as a neuroprotective factor in developing neurons. Therefore, we tested the hypothesis that ethanol targets TERT transcription in progenitor neuroblasts. Reverse transcription PCR (Figure 8A, B) shows that TERT mRNA levels were modestly but statistically significantly increased (p < 0.05) at the low ethanol dosage (120 mg/ml). Conversely, high levels of ethanol (620 mg/dl) produced a statistically significant decrease in TERT mRNA levels. We hypothesized that the ethanol-induced changes in TERT mRNA levels would lead to changes in the activity of telomerase complex itself. However, quantitative measurements of telomerase activity indicate that low and high doses of ethanol do not affect the activity of the telomerase complex (Figure 8C). The cycle threshold values in the low and high ethanol-treated groups were identical, reflecting equal amounts of telomerase activity in each sample. The increase in TERT transcription following low doses of ethanol (120 mg/dl) was not followed by a measurable increase in telomerase activity, an unexpected observation in light of the marked induction of DNA synthesis and cell-cycle progression illustrated in Figure 2A, B. These results suggest that ethanol may uncouple telomerase activation from neuroepithelial cell proliferation.

### Ethanol prevents subsequent differentiation of cells derived from neurosphere cultures

**Figure 9:**
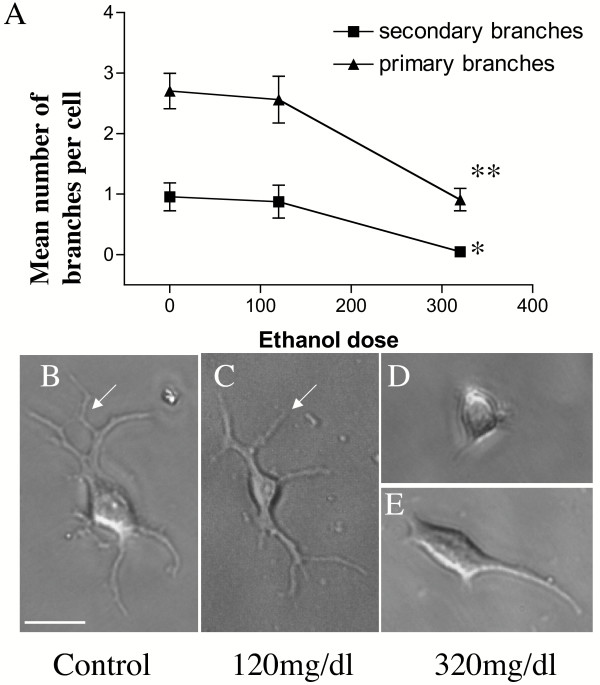
Pre-treatment with ethanol disrupts retinoic acid-mediated differentiation. Neurosphere cultures were exposed to a dose range of ethanol for 4 days, dissociated into a single cell suspension, plated and then differentiated with retinoic acid (**A**) Quantitative analysis of the mean number (± SEM) of primary and secondary branches per cell, formed in the presence of retinoic acid, as a function of ethanol pre-treatment dose. Asterisks indicate statistically significant differences from non-ethanol exposed, control cultures, * = p < 0.003; ** = p < 0.0001. (**B-E**) Representative photomicrographs of branching patterns observed in the presence of retinoic acid, in control (**B**), low (**C**) and moderate doses (**D,E**) of ethanol. Arrows point to second-order branches, which are missing in cells derived from neurosphere cultures exposed to 320 mg/dl ethanol. Scale bar, **B-E**, 25 uM

In the final set of experiments, we examined the effect of ethanol on the subsequent differentiation potential of cortical progenitor cells. We hypothesized that ethanol induction of cell proliferation, would alter the ability of cortical progenitors to differentiate into neurons. Rat neurosphere cultures we maintained in control medium or exposed to the low or moderate doses of ethanol (120 or 320 mg/dl) for 4 days. Following this period of ethanol exposure, we dissociated neurospheres and cultured the constituent cells at low density in the presence of 10 nM retinoic acid. Neurosphere-derived cells were cultured at low density so that individual cells would have a low probability of contacting another cell. This protocol was followed to reduce the impact of cell-cell interactions and target-derived trophic support mechanisms on neuronal differentiation. We selected retinoic acid as the differentiation stimulus, since previous work in our laboratory showed that retinoic acid is a strong trigger for cortical neuroepithelial differentiation [54]. Overall, ethanol pre-exposure led to a significant dose-related decline in the number of first-order (p < 0.0001) and second-order (p < 0.003) branches that were induced following retinoic acid exposure (Figure 9A). Naïve cells, and cells derived from neurospheres pre-exposed to the low dose of ethanol (120 mg/dl) exhibited a strong differentiation phenotype in response to retinoic acid (Figure 9B, C), including the expression of second-order neurite branching. However, cells derived from neurospheres pre-exposed to 320 mg/dl exhibited a marked reduction in neurite branching in response to retinoic acid (Figure 9D).

## Discussion

Our data show that ethanol did not induce appreciable apoptosis in embryonic cortical-derived neurosphere cultures. These data are somewhat surprising because the activation of cell death mechanisms is intuitively consistent with ethanol's adverse effects on brain development. However, ethanol induction of cell death may be differentiation stage-specific. For example, previous research in our laboratory demonstrated that ethanol concentrations equal to those used in this study induced apoptosis in more differentiated postnatal cortical [10], and cerebellar explant cultures [11], perhaps because tumor-suppressor genes like p53, and down-stream pro-apoptotic genes like Bax are suppressed in proliferating cortical cells and only induced during differentiation [54]. Clearly, more research is needed to identify genes and mechanisms that confer apoptosis-resistance to precursors, but not differentiated neuronal cells.

### Ethanol promotes cell-cycle activity

Rather than killing cells, ethanol stimulated DNA synthesis and promoted cell-cycle progression in cerebral cortical precursors as indicated by the increase in the size variation and number of neurospheres, induction of S-phase, and increased progression through G2/M phases of the cell cycle. On the other hand, ethanol induced asymmetric division in progenitor cells and decreased the cell surface expression of a number of stem cell markers, i.e., c-kit/CD117, Sca-1 (Ly6A/E), CD133/prominin-1 and the ABCG2 transporter. In contrast to the induction of cell cycle, asymmetric cell division and loss of stem cell antigens are both indicative of neuroepithelial maturation. These apparently antagonistic data are best explained within the context of normal cerebral cortex development. Neurogenesis in the developing cerebral cortex, occurs within two distinct germinal zones, the earlier developing ventricular zone, and the later developing subventricular zone. Proliferation within the ventricular zone serves to replenish stem and blast pools (by symmetrical division) and to generate more mature, fate-restricted daughter progeny (by asymmetrical division, [55]). The ethanol-induced asymmetric division and loss of stem cell markers supports the notion that ethanol induces maturation of the stem cell pool. The daughter blast cells or 'proto-neurons' that exit the ventricular zone, migrate in turn to the subventricular zone where they may continue to proliferate and generate neurons, principally by symmetrical division [55]. The increased proliferation that we observed in ethanol-treated cultures may therefore reflect the expansion of a 'subventricular-zone-like phenotype'. Recent evidence suggests that the first blast cells that migrate out of the ventricular zone are in fact radial glia, and that these cells are also precursors for mature neurons (for review, see [56]). This view is consistent with our observations that asymmetric division occasionally resulted in the appearance of polarized cells with long (radial-like) processes that fit morphological criteria for radial glia. Further support for this 'two-zone' hypothesis of ethanol's action comes from our observations that ethanol treatment does ultimately deplete the regenerative capacity of cortical neuroepithelial precursors. Cells derived from ethanol-pretreated neurospheres exhibited a decreased ability to form new colonies, implying that the loss of stem cell markers does indeed mean that ethanol depletes stem cells from the pool of immature precursors.

Overall, this interpretation of these data in terms of stem cell maturation is also consistent with previously published work from other laboratories using alternate labeling indices, showing that ethanol decreases cell proliferation within the ventricular zone, while increasing cell proliferation within the more differentiated subventricular zone [27,57,58]. Increased cell proliferation is a requirement for differentiation in other tissues as well. For example, in the hematopoietic system, the differentiation of CD34+ stem cells into pro-erythroblasts is accompanied by a significant increase in cell cycle. This induction of cell cycle supports successive stages of blast maturation, till the formation of the orthochromatophilic erythroblast, the immediate blast precursor to the differentiated erythrocyte [26].

The consequences of enhanced proliferation on neural differentiation remain to be determined. The diversity of cellular subpopulations that contribute to the lamination of the cortical plate is likely to be disrupted, leading to an overabundance of some neuroblast populations at the expense of others. Such population imbalances may be one cause of phenomena like cellular heterotopias that have been observed in brains of children with FAS [59], and in animal models of FAS [60]. While *in vitro *models suggest that migration defects contribute to the formation of heterotopias [61], the role of aberrant ethanol-driven expansion of specific progenitor pools in heterotopia formation cannot be ruled out.

### Ethanol limits cortical stem cell diversity in neurosphere cultures

Neurosphere cultures consist of a heterogeneous mixture of multi-potent neuronal stem cells and daughter neuroblasts [47]. CD133/prominin-1 identifies stem cell groups with multi-potent properties in the developing central nervous system of humans and rodents [62-66]. Sca-1 (Ly6A/E) identifies hematopoietic cells with the potential to form neurons, while CD117/C-kit [67-69] and the ABCG2 multi drug-resistance transporter [48] are expressed in stem cell precursors of the rodent central nervous system. We demonstrate that populations of cortical precursors do express these stem cell markers. Nestin mRNA was also expressed in cultured cortical precursors, but nestin mRNA levels did not change with ethanol exposure, indicating that despite maturational pressure, immature, possibly blast-type cells continue to persist in ethanol-treated cultures. However nestin mRNA expression is not informative about the extent to which ethanol affects the heterogeneity of stem cells in culture, because nestin expression is a common phenotype of stem and more differentiated blast cells. In contrast, ethanol significantly decreased the numbers of cells that expressed cell-surface Sca-1, c-kit, CD133 and ABCG2. The absence of an appreciable degree of apoptosis in ethanol-treated cultures, suggests that ethanol influences the diversity of the stem cell pool, rather than cell survival *per se*. In the context of the previously noted requirement for cell cycle induction as a component of stem cell to blast cell transformation [26], it is interesting to note that the suppression of the stem cell marker Sca-1 [70]) promotes myoblast proliferation. Consequently, our observations that ethanol induces cell cycle and decreases the expression of stem cell markers is mutually consistent with the hypothesis that ethanol drives stem cell to blast cell transformation in cortical neuroepithelial precursors.

The functions ascribed to the stem cell markers that we utilized in this study are particularly relevant to the issue of ethanol's impact on cell fate determination. For example, CD133 and c-kit are important components of lipid raft micro-domains [71,72], that sequester cell-signaling machinery. In bone marrow, Stem Cell Factor/c-kit interactions form a critical component of stromal-driven differentiation of several hematopoietic lineages [73]. Therefore, changes in the composition of lipid rafts are likely to result in significant alterations to signaling mechanisms that drive stem cell differentiation, and in the brain, lead to aberrant development. Sca-1 is also a signaling molecule, linked to the cell-surface by a phosphatidyl-inositol anchor, and is important for stimulating hematopoietic stem cell renewal [74]. Sca-1 expression is suppressed in hematopoietic stem cells, during the process of lymphocyte differentiation [75]. While Sca-1 may well exhibit different developmental-stage associated kinetics in the neuroepithelium, ethanol-dependent depletion of Sca-1 in the neuroepithelium may similarly drive precursor maturation and prevent the renewal of a stem cell pool. Interestingly, both Sca-1 [70] and CD117/c-kit [76,77] share the Src-like tyrosine kinase *Fyn *as a common signaling intermediary, suggesting that the two cell-surface molecules mediate common functions during development. *Fyn *in turn, mediates ethanol-sensitivity and dependence in the adult animal, and a *Fyn *polymorphism is predictive of alcohol dependence in human populations [78-80], further suggesting that ethanol's effects in the developing and adult brain are likely to be mediated by common mechanisms. Finally, ethanol suppression of the expression of multi-drug resistance transporters like ABCG2 is also likely to be clinically significant. Multi-drug resistance transporters are thought to protect stem cells from damage by non-selectively extruding a wide variety of cytotoxic compounds [52]. Ethanol exposure, by suppressing ABCG2 expression, may decrease the survivability of neural stem cells in the face of subsequent toxic insults, including perhaps, subsequent episodes of ethanol exposure.

### Ethanol's effect on Telomerase activity and TERT transcription

Rapidly dividing tissues are susceptible to errors in DNA synthesis that can lead to DNA damage, chromosomal instability and cell death. The telomerase complex maintains chromosomal ends, and consequently ensures chromosome stability during DNA synthesis (reviewed in [81-83]). Telomerase activity is robust during embryogenesis [84] and is particularly elevated in the proliferating neuroepithelium. Though activity is decreased following cellular differentiation, the catalytic component of telomerase (TERT) continues to be expressed widely in post-mitotic neurons where it appears to function as a neuroprotective factor [40,41,85]. The lower dose of ethanol (120 mg/dl) resulted in increased TERT mRNA levels, perhaps as a compensatory protective response. In contrast, the suppression of TERT mRNA expression at high doses (620 mg/dl) is inconsistent with the observed increase in cell cycle progression at that dose. Interestingly, TERT physically associates with the anti-apoptotic kinase AKT [86], and a reduction in TERT expression levels has the potential to render dividing cortical precursors susceptible to future apoptotic stimuli, (for example, see [41,87,88]), even if ethanol itself does not induce apoptosis.

Because we observed that ethanol increased cell cycle activity in neurosphere cultures, we hypothesized that ethanol would also increase telomerase activity. Surprisingly, ethanol had no effect on telomerase activity. The lack of coordination between telomerase activity and changes in cell cycle activity suggests that DNA synthesis is likely to be incomplete in telomeric regions, increasing genomic instability in proliferating, ethanol-exposed neuroblasts. Recent research indicates that the prenatal rodent neuroepithelium normally exhibits a significant degree of genetic mosaicism in which 33% of all cortical precursors display some degree of chromosomal aneuploidy [89] and loss of heterozygosity [90]. It is surprising that the developing neuroepithelium tolerates such a significant degree of genetic instability without negative sequellae. However, given a high tolerance for mosaicism, any increases in genetic instability among ethanol-exposed cells of the neuroepithelium may not result in apoptosis, but in altered patterns of gene expression due to mechanisms like gene shedding [90], consequently altering neural differentiation. An intriguing possibility, supported by the discrepancy between telomerase and cell cycle data, is that the accumulation of progenitors with greater than G0 DNA content in ethanol-treated cultures represents increased aneuploidy rather than increased progression through cell cycle *per se*.

### Ethanol pre-treatment prevents stimulus-dependent neuronal differentiation

The loss of stem cell markers, and evidence for increased cell division led us to hypothesize that ethanol exposure would promote the further maturation of the cortical stem and blast cells. However, contrary to our hypothesis, ethanol pre-treated neural progenitor cells were refractory to retinoic acid stimulation. Though retinoic acid is a potent stimulator of neuronal differentiation [54], it appears to have growth-stimulatory effects in part, by stimulating neurotrophic signaling *via *factors like glial-derived neurotrophic factor and neurotrophin-3 [91]. Since ethanol has been previously shown to interfere with growth factor signaling mechanisms in a variety of neural differentiation models [11,14-17,92], it is likely that ethanol disrupts growth factor signaling to interfere with the response to retinoic acid as well. The developmental consequences of delays in neuronal differentiation are likely to be profound. Even if the effect of ethanol is transient, the inside-out lamination of the cortical plate is likely to be significantly disrupted, because later-generated neural precursors do not appear to be able to populate earlier-developing cortical plate laminae, after the critical period for the generation of that specific lamina has been passed [93].

## Conclusion

*In toto*, our data support the hypothesis that the elimination of neural stem cell diversity contributes to the etiology of prenatal brain damage caused by maternal ethanol consumption. Rather than inducing apoptosis, ethanol stimulates cell-cycle activity and eliminates stem cell antigen expression. It is likely therefore, that ethanol drives the stem cell to blast cell transformation, ultimately depleting the reserve regenerative capacity of cerebral cortical neuroepithelium. Secondly, the uncoupling of telomerase activity from cell-cycle induction suggests that ethanol exposure may hasten telomere decay and cellular senescence, rendering maturing neuroblasts susceptible to genetic damage as they proceed through cycles of maturation-driven proliferation. Finally, if the ventricular and subventricular zones are indeed 'proto-maps' of the mature cortical plate [28], restrictions in the diversity of the stem cell pool are likely to translate into loss of diversity of neuronal and glial phenotypes that populate the mature cortical plate. While the developmental consequences of perturbing the cortical stem cell pool have not been adequately assessed in experimental systems of ethanol-induced brain damage, one likely sequel to such perturbations is a permanent transformation in the phenotypic composition of the neural network of the cerebral cortex.

## Methods

### Isolation of embryonic neural precursors

The University Laboratory Animal Care Committee approved all animal procedures. Timed-pregnant Sprague-Dawley rats (gestational day [GD] 13) were purchased from Harlan, Houston, Texas, and maintained in the animal housing facility at Texas A&M University System Health Sciences Center, College of Medicine for two days until the pregnancy matured to GD15. At GD 15, pregnant females were anesthetized with ketamine (0.09 mg/gram)/xylazine (0.106 mg/gram) by intraperitoneal injection. Under aseptic conditions, the gravid uterus was delivered through a midline transverse abdominal incision. The gravid uterus was rinsed in chilled PBS containing 1% penicillin/streptomycin. Eight to ten fetuses were isolated and rinsed three times in chilled PBS. Anesthesia was achieved by placing fetuses in ice-cold Gey's Balanced Salt Solution. Fetuses were rapidly decapitated, and whole brains were removed and placed in chilled Gey's Balanced Salt Solution supplemented with glucose and magnesium chloride. Brains remained suspended in this solution throughout the remainder of the microdissection procedure. Meningeal tissue was removed, regions of the rat fetal brain corresponding to the structural precursor of the neocortex were isolated, and care was taken to exclude the structural precursors to the striatum and hippocampus. Individual cortical fragments were collected in sterile 15 ml conical tubes and gently triturated in trypsin/EDTA. Trypsin was inactivated with DMEM containing 10% fetal bovine serum. The cell suspension was centrifuged for 5 minutes at 18°C, 1000 rpm (300 × g). Cell pellets were resuspended in chilled PBS containing 0.5% BSA, Fraction-V, (#1526037, Invitrogen) and 2.0 mM EDTA. Total cell counts were determined using a hemocytometer

### Cell culture

Neurosphere cultures were established from acutely dissociated cortices (described above). Precursor cultures were established at an initial density of 10^6 ^cells in T-25 flasks containing 5–6 ml of serum-free mitogenic media (DMEM/F12 (#11330-032 Invitrogen), 20 ng/ml bFGF (#13256-029 Invitrogen), 20 ng/ml hEGF (#53003-018 Invitrogen), ITS-X supplement (#51500-056 Invitrogen), 0.85 Units/ml heparin (#15077-019 Invitrogen), and 20 nM progesterone (#P6149 Sigma)). Cultures were incubated at 37°C, 5% CO_2 _in a humidified environment for 48–72 hours before the commencement of ethanol treatments to allow for stabilization of culture and for the generation of small neurospheres, a sign of precursor expansion. For some experiments, following ethanol treatment, neurosphere cultures were dissociated and differentiated for 4.5 days in the presence of 10 nM retinoic acid (in DMEM/F12 and 1%N2 supplement).

### Ethanol treatment

Cultures were assigned to three treatment groups: (I) a control group containing no ethanol; (II) a low dose group containing 120 mg/dl (26.07 mM); (III) a high dose group containing 620 mg/dl (134.78 mM, prepared from 95% ethanol, molecular biology grade-Sigma). For some experiments, an intermediate, dose of ethanol, 320 mg/dl (69.56 mM) was used in place of the high dose. Gas chromatographic analyses indicated that the low and high doses resulted in ethanol levels of 97–106 mg/dl (21.09 mM–23.04 mM) and 367–557 mg/dl (79.35 mM–121.09 mM) respectively, while the moderate dose of ethanol resulted in measured levels between 182–227 mg/dl (39.57 mM–49.35 mM). These concentrations are within the range that has been previously observed in chronic alcoholics [94,95]. Doses of 150 mg/dl and 200 mg/dl have been used previously in cell culture models of chronic ethanol exposure [96,97]. The ethanol concentrations used in this study are expected to reflect the levels in the fetus during prenatal exposure, as it has been shown in rodent models that maternal blood alcohol levels produce equivalent concentrations in the fetus [98]. The ethanol treatment lasted 4 days. Ethanol containing media was replaced daily throughout the duration of the treatment period.

### Labeling for stem cell surface antigen expression

Neurosphere cultures were dissociated in Accumax solution (Innovative Cell Technologies) by gentle trituration through a fire polished pipette. Precursors were labeled with phycoerythrin-conjugated antibodies to Sca-1/Ly6A/E (1 ug, Caltag Labs, MM4104), CD117/c-kit (1 ug, BD Pharmingen, 555714), CD133/prominin-1 (0.55 ug, Miltenyi Biotec, AC141), BCRP1 (ABCG2)-FITC (1 ug, Chemicon International MAB4155F) or isotype-matched IgG. 10^6 ^live cells were incubated separately with each of the above antibodies for 10 minutes at 4°C and immediately analyzed by flow cytometry. In each group, an equal amount of unlabeled IgG isotype-matched antibody was added to the labeling solution to serve as a blocking reagent for potential Fc-Receptor sites on the cell surface. Between 10,000 and 100,000 cells were counted in each sample. Staining levels from cells labeled with phycoerythrin-conjugated IgG isotype-matched antibodies served as a measure of background fluorescence. This background was subtracted from all groups labeled with stem cell antigen markers and flow cytometric data were collected for this population of cells. The proportion of labeled cells was expressed as the percentage of total cells gated.

### Propidium iodide staining for DNA content

Neurosphere cultures were dissociated in Accumax solution (Innovative Cell Technologies) as mentioned above. Dissociated cells were collected by brief centrifugation at 1000 rpm, 18°C and resuspended in cold PBS. An equal volume of 2% phosphate-buffered paraformaldehyde was added to yield a final concentration of 1% paraformaldehyde. Cells were fixed for 45 minutes at 4°C. Fixed cells were washed twice in PBS and resuspended in PBS containing 0.5 mM EDTA, 0.1% Triton X-100, 0.05 mg/ml RNAse A (#R4642 Sigma). Cells were incubated at 37°C for 30 minutes to allow for RNA degradation. Propidium iodide (#1348639 Roche) was added to the solution at a final dilution of 1:50 and incubated for at least 30 minutes at 4°C. At least 10,000 cells were analyzed by flow cytometry.

### Flow cytometry

Cell cycle analysis and measurements of stem cell antigen levels were conducted on a FACS Calibur Flow Cytometer (Beckton Dickinson). Excitation wavelength was set at 488 nm (Argon laser) and emission spectra for phycoerythrin and propidium iodide were 575 nm and 630 nm, respectively. Cell-cycle histograms were generated using Cell Quest software for Macintosh.

### Immuno-fluorescence analysis

Neurosphere cultures were assayed for the expression of the neuroepithelial marker Nestin, and neuronal marker NeuN (neuronal nuclear antigen), according to previously published protocols [54]. After 3.5–4 days, media was removed and cultures were washed in PBS, fixed in 1% phosphate-buffered paraformaldehyde for 45 minutes at room temperature. Cultures were rinsed twice with PBS and once with TBS, followed by incubation in blocking solution (2% normal serum, 0.1% BSA, 0.2% triton x-100, in TBS) for 1 hour at room temperature. Antibodies (all from Chemicon) against nestin 1:100 (MAB3353) and NeuN 1:100 (MAB377), were diluted in staining solution (TBS 0.1% BSA), and incubated with cells overnight at 4°C. Following three washes in TBS, cells were labeled with rat-adsorbed, biotinylated secondary horse anti-mouse antibodies (1:250) (Vector Laboratories) in TBS according to manufacturers instructions, and antibody binding was visualized with by conjugation with avidin-rhodamine 1:250 (Vector Laboratories). Cells were mounted in Fluorescence Mounting Media containing DAPI (Vector Laboratories).

### RNA extraction and cDNA synthesis

RNA was extracted using the Trizol reagent in accordance with the manufacturer's instructions (Invitrogen). RNA was purified with the SV Total RNA Isolation System (#Z3100 Promega). 2.5 micrograms of total RNA was used to synthesize cDNA using the Superscript III First-Strand Synthesis System for RT-PCR (Invitrogen #18080-051) and random hexamers according to the manufacturer's protocol.

### Polymerase chain reaction (PCR)

PCR was performed by conventional thermocycling methods. 2 uls of cDNA mixture from above was combined with the primers (200 nM each) and PCR Supermix (#10572-014, Invitrogen) in a total volume of 50 ul. Primers for TERT were those used in (Holzmann et al., 2003). PCR products were resolved by agarose gel electrophoresis and visualized by ethidium bromide staining. The cyclophilin-A (Peptidyl-prolyl cis-trans isomerase A) gene served as an internal control measure [99,100]. Forward and reverse primer sequences are as indicated in Table 1.

### Measurement of telomerase activity by TRAP Assay

Telomerase is a reverse transcriptase ribonucleoprotein complex that catalyzes the addition of six base pair repeats to telomeric ends [101-103]. The PCR-based Telomeric Repeat Amplification Protocol (TRAP), to measure telomerase activity in biological samples, was adapted from [38], as described in [104], based on SYBR-Green I fluorescence and real-time PCR. PCR reactions were performed with SYBR Green JumpStart TaqReady Mix (Sigma #S4438). Cell pellets were homogenized in 200 uL of cold lysis buffer and chilled on ice for 30 minutes, then re-sedimented by centrifugation at 16,000 × g for 20 minutes at 4°C, then lysed in CHAPS lysis buffer [85]. Lysates were quantified for total protein content and diluted to a working concentration of 100 ng/ul and stored at -80°C until use. The TS and ACX primers used for both the telomerase elongation and the amplification of telomerase elongation products are as described in [105]. The following components were mixed in a 25 ul volume and analyzed for telomerase activity; 13 ul SYBR Jumpstart Mix, 0.1 ug (1 ul) TS primer, 0.05 ug (1 ul) ACX primer (for primer sequence, see Table 1), 0.2 ul T4 Gene 32 Protein (1 unit, Roche 972983), 1 ul extract (100–500 ng) and water to 25 ul final volume. SYBR Green fluorescence intensity was quantified during the 60°C annealing/elongation step and expressed graphically as a function of cycle number. Cycle threshold values (Ct) are an indirect measurement of telomerase activity and represent the cycle number at which the generated product reaches a preset threshold level. Thresholds were set at 10 standard deviations above background fluorescence.

### Data analysis

TERT and nestin mRNA levels were quantified by densitometric measurement, and were expressed as a ratio of cyclophilin-A mRNA expression (Molecular Analyst for Windows, BioRad). For TRAP assays, Sybr-Green fluorescence intensities were plotted against cycle number, and cycle threshold (ct) determined. Neurosphere number and size were computed using a Java version of NIH image (ImageJ, V1.32j (NIH)). Data were analyzed using a standard statistical package, SPSS for Windows (Version 11). Analysis of Variance (ANOVA) and the Fischer's Least Significant Difference post-hoc test were used to identify statistically significant differences between groups. Alternatively, Pearson's correlations, and two-tailed tests of significance were computed to determine relationships between ethanol dose and response. Statistical significance was set at p < 0.05.

## List of abbreviations

CD117 C-kit

CD133 prominin-1

ABCG2 ATP-binding cassette, sub-family G (WHITE), member 2; member of the ATP-dependent multi-drug resistance transporter family; aka. BCRP1

FAS Fetal Alcohol Syndrome

NeuN Neuronal nuclear antigen

Sca-1 Stem Cell Antigen-1 or Ly6A/E

TERT Telomerase reverse transcriptase subunit

## Authors' contributions

DRS and RCM contributed to the design of the experiments, data analysis and manuscript preparation. DRS, LSK, TLP, CC and JDT contributed to the cell culture, immunofluorescence and flow cytometric analyses. DRS and JDT contributed the RT-PCR and telomerase activity assay. All authors read and approved the final manuscript.

## Figures and Tables

**Table 1:** List of PCR primers

**Primer Name**	**Sequence**	**Product Size**	**Accession #/Reference**
TS primer	5'-ATTCCGTCGAGCAGAGTT-3'		[105]
ACX primer	5'-GCGCGG [CTTACC]_3_CTAACC-3'		
R-Tert_Forward	GGTCTTCCGCACGTTGGTTG	349bp	[106]
R-Tert_Reverse	CAGCAGGTAGAGCGCACAGT		
Rat_Nestin_Forward	TGCAGCCACTGAGGTATCTG	1061bp	Acc#: M34384
Rat_Nestin_Reverse	AGTTCCCACTCCTGTGGTTG		
Cyclophilin A_Forward	TGGTCAACCCCACCGTGTTCTTCG	372bp	Acc#: M19533
Cyclophilin A_Reverse	TGCCATCCAGCCACTCAGTCTTGG		
